# Geodesic-Based Method for Improving Matching Efficiency of Underwater Terrain Matching Navigation

**DOI:** 10.3390/s19122709

**Published:** 2019-06-16

**Authors:** Zhaowei Li, Wei Zheng, Fan Wu

**Affiliations:** 1 Qian Xuesen Laboratory of Technology, China Academy of Space Technology, Beijing 100094, China; lizhaowei@qxslab.cn; 2 School of Geomatics, Liaoning Technical University, Fuxin 123000, China; 3 School of Surveying and Landing Information Engineering, Henan Polytechnic University, Jiaozuo 454000, China; 4 HuaiHai Institute of Technology, School of Geomatics and Marine Information, Lianyungang 222005, China; 5 State Key Laboratory of Geodesy and Earth’s Dynamics, Institute of Geodesy and Geophysics, Chinese Academy of Sciences, Wuhan 430077, China

**Keywords:** geodesic-based method, matching efficiency, underwater terrain matching navigation, search matching time, terrain suitability

## Abstract

In this study, we improved the matching efficiency of underwater terrain matching navigation. Firstly, a new geodesic-based method was developed by combining the law of the shortest arc in spherical geometry with the theory of the attitude control in space and maritime environments. Secondly, along a design track, the geodesic-based method helped reduce the radius of the search matching area, and improved the matching efficiency. Finally, for parameter setting, the search matching time of underwater terrain matching navigation was reduced from 9.84 s to 1.29 s (about 7.6 times), with the matching accuracy being invariable using the new geodesic-based method.

## 1. Introduction

The aerospace-aeronautics-marine integration of marine navigation via multi-source information and technical means is a major trend in the development of underwater vehicle navigation systems. At present, underwater vehicle navigation systems are mainly composed of an inertial navigation system (INS). However, the INS error accumulates over time. Therefore, to compensate for the accumulative error of INS and to ensure the navigation security and the strike accuracy, some externally-aided methods have been used for periodic readjustment and correction [1,2,3,4].

**Figure 1:**
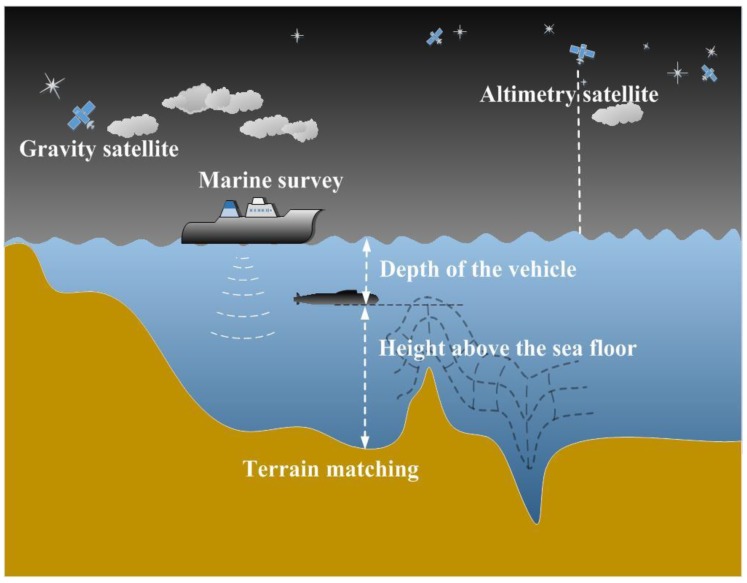
Schematic diagram of underwater terrain matching navigation.

The geophysical field is united with the INS to form the passive navigation system to solve the positioning error of INS that accumulates over time. At present, the underwater technologies for long-term hidden navigation can be aided by terrain, gravity, and geomagnetism. Due to the long- and short-term variations in the geomagnetic field, it is difficult to obtain a high-precision geomagnetic map. Additionally, magnetic measurement has its own limitations, such as magnetic interference [5]. The gravity field [6,7,8,9,10] and terrain are the main technical means of auxiliary navigation. Terrain has been studied previously, and terrain matching navigation has been used for decades in land and air equipment, e.g., in aircraft and cruise missiles. This technique also has been applied in underwater vehicles, as shown in Figure 1. In the research within the development strategy “Technology for the United States Navy in 2000–2035”, the goal of improving the accuracy of underwater vehicle navigation using terrain matching technology was introduced [11,12,13].

The core problem of terrain matching navigation technology is creating the technology needed, such as high-precision and high-resolution global seabed digital terrain maps, high-precision bathymetric measurements, and terrain-matching algorithms. Since the end of the last century, many matching algorithms have been proposed [14,15,16,17,18,19]. According to the algorithm principle, terrain-aided navigation can be divided into three types. The first technique is correlation analysis, which is realized by calculating the correlation of a set of in-situ-collected terrain height data with different sets of terrain heights taken from the underwater digital terrain map. The second technique is the extended Kalman filter (EKF), and the last technique is a method based on the probability criterion. The Terrain Contour Matching (TERCOM) algorithm is popular among them. Its advantages are its simple positioning and high positioning accuracy. Its shortcoming is that the navigation error increases sharply when the course has a large deviation. This algorithm has a high computational complexity and low operational efficiency [20,21,22,23]. Therefore, determining how to improve the matching efficiency and positioning accuracy of the TERCOM algorithm is a research hotspot in the field of underwater navigation. By introducing the idea of forecast control, Xu et al. proposed a terrain contour forecast matching method based on height features. The calculation amount was reduced by about 35%, and the real-time performance of the navigation system was improved [24]. Yu et al. designed a TERCOM algorithm based on image layered-searching. The distinguishing sequence algorithm in image layered-searching was applied to accelerate TERCOM by dividing the terrain map to three layers. A comparing logic was constructed between the second and third layer. By this means, matching speed increased and false matching decreased. Simulation results showed that the matching efficiency increased 93% [25]. Wang et al. designed a new method based on the integration of TERCOM and Iterative Closest Contour Point (ICCP) technology. Then, they analyzed the rough matching algorithm and its performance, and studied the accuracy of the matching algorithm using the improved ICCP matching algorithm. After an experiment in Bohai Bay, the matching efficiency of this new algorithm increased about two to six times [26].

Differing from the previous studies, the aim of this study is to improve the matching efficiency of underwater navigation. A new geodesic-based method is proposed to improve the matching efficiency of underwater terrain matching navigation with invariable accuracy. This method helps to accurately adjust heading deviation, and improves the matching efficiency by reducing the radius of the search matching area.

## 2. Materials and Methods

**Figure 2:**
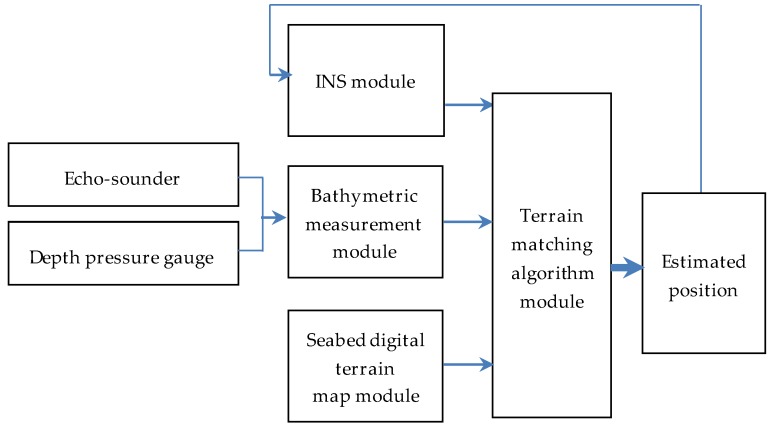
Configuration of underwater terrain matching navigation system.

The composition of the basic module of the underwater terrain matching navigation system is shown in Figure 2 [27,28], including the INS module, the seabed digital terrain map module, the bathymetric measurement module, and the terrain matching algorithm module. The INS module provides the key navigation information. The seabed digital terrain map module is usually represented by a digital bathymetric map. The bathymetric measurement module provides the depth at certain points on the sea floor relative to a defined vertical datum. There are numerous vertical datums, with Mean Sea Level (MSL) being the most widely used. The depth of the underwater vehicle is measured by a depth pressure gauge, and the height of the underwater vehicle relative to the sea floor is measured by an echo-sounder. The terrain matching algorithm module is designed to use a set of bathymetric measurements to build a terrain profile, and then find an optimal position estimate by comparing it with a prior map database.

The performance of the underwater terrain matching navigation system is closely connected with the terrain matching algorithm and sea floor characteristics. The existing matching algorithms, such as TERCOM and ICCP, are batches oriented based on correlation analysis. Sandia Inertial Terrain-Aided Navigation (SITAN) and particle filter (PF) are matching algorithms that are based on the extended Kalman filter (EKF) and probability criterion, respectively. The characteristics of the sea floor are usually described using terrain feature parameters. According to different criterions, some terrain feature parameters have been defined, including standard deviation, entropy function, roughness, and correlation length, which respectively reflect terrain fluctuation, uncertainty, local variety, and correlation characteristics of adjacent areas. The purpose is to construct a discriminant analysis model of terrain suitability using terrain feature parameters, which is important for the selection of the terrain matching area [29,30,31,32,33,34].

**Figure 3:**
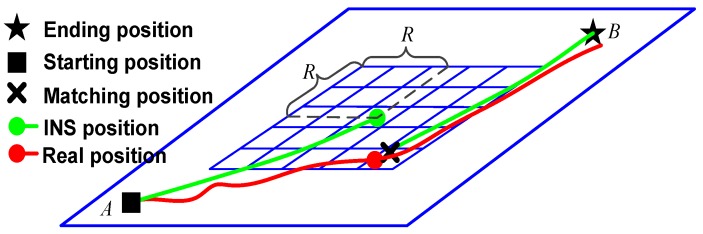
Algorithm flowchart of underwater terrain matching navigation.

The TERCOM algorithm belongs to the class of search area methods. The best position estimation is determined by an exhaustive search terrain database [35]. The selected study area was pre-validated in this experiment, and was found to be suitable. We assumed that the navigation task covers from position A to position B. Figure 3 shows that the algorithm flowchart of underwater terrain matching navigation is based on the geodesic-based method.

Step 1: The aim is to determine the coordinates of the starting position A and the ending position B, and plan the route.

Step 2: The positioning of an underwater vehicle depends on the INS before entering the matching area. According to the relative position of the current INS and the ending position B, the heading is adjusted at every fixed time interval (T) using the geodesic-based method.

Step 3: After entering the matching area, the terrain matching is performed by the TERCOM algorithm if a set of sampled data is long enough. Then, the positioning error of INS is revised on the basis of the matching result. For example, the search area can be ±3σ around the INS estimation.

Step 4: Steps 2 and 3 are repeated until the ending position B is reached.

**Figure 4:**
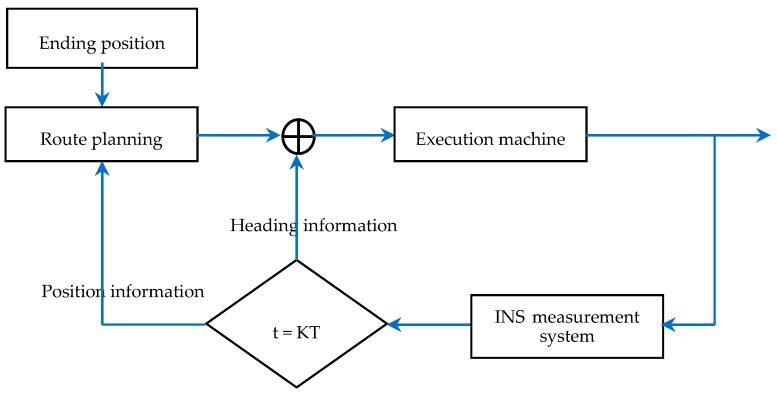
Structure diagram of the geodesic-based method.

**Figure 5:**
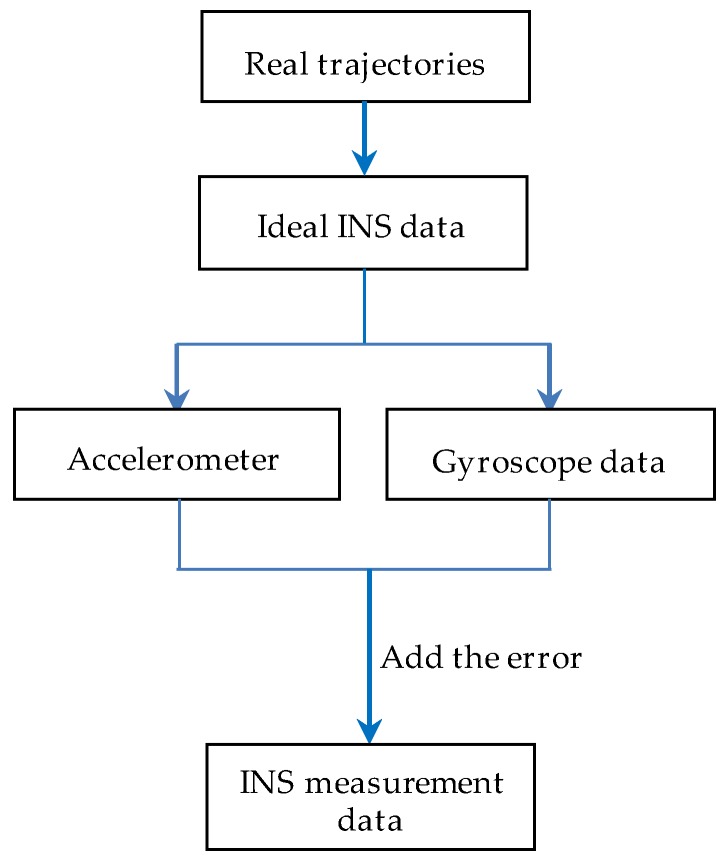
Flowchart of INS data acquisition.

Figure 4 shows the structure diagram of the geodesic-based method, where the transfer function of the execution machine is taken as 1 and K is a natural number. The INS measurement data were obtained following the method shown in Figure 5.

The route planning is based on the principle of the shortest distance, and the Earth’s spherical factor is also considered. On a planar plane, the shortest distance between point A and B is a straight line. However, on the standard unit sphere, the shortest distance between two points is a great circle arc [36].

**Figure 6:**
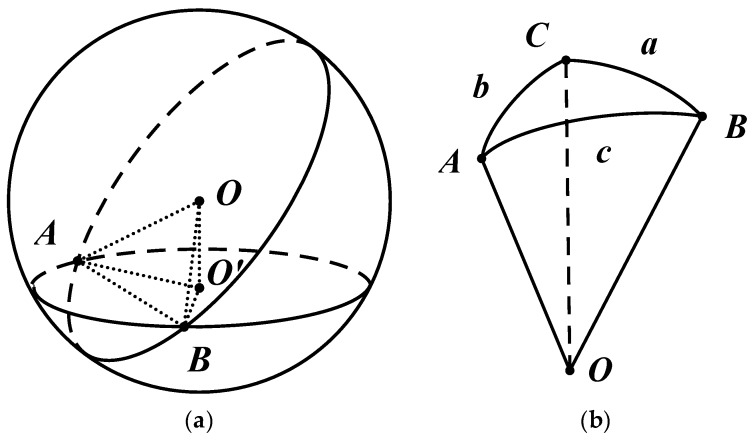
Theory of the shortest spherical arc: (**a**) unit sphere; and (**b**) relationships between sides and angles of a spherical triangle.

We assumed that the earth is a standard unit sphere, as shown in Figure 6a. The shortest distance between point A and B is a great circle arc corresponding to the center of the circle rather than the arc on the latitudinal coil.

As shown in Figure 6b, in the northern hemisphere, we assume that the point C is the North Pole, < lat_A, lon_A > is the latitude and the longitude of the current INS position, and < lat_B, lon_B > is the latitude and the longitude of the ending position B. The calculation formula of spherical triangle is:

(1) sinCcotA=cotasinb−cosCcosb

The dihedral angle C can be obtained by the longitudinal difference between two points A and B:

(2) C=(lon_B−lon_A)pi/180

where *a* and *b* are the circle arcs corresponding to the center angle ∠COB and ∠COA, respectively, which can be obtained from the latitudes of two points A and B:

*a* = (90−*lat*_*B*)*pi*/180 (3)

(4) b=(90−lat_A)pi/180

The optimal heading angle can be calculated based on the principle of the shortest distance using Equations (1) to (4), and the value of heading angle represents the angle of the north by east:

(5) $$A=arc\cot\frac{\left(\cot a\sin b-\cos C\cos b\right)}{\sin C}$$

## 3. Results

### 3.1. Numerical Simulation and Verification of the Geodesic-Based Method

**Figure 7:**
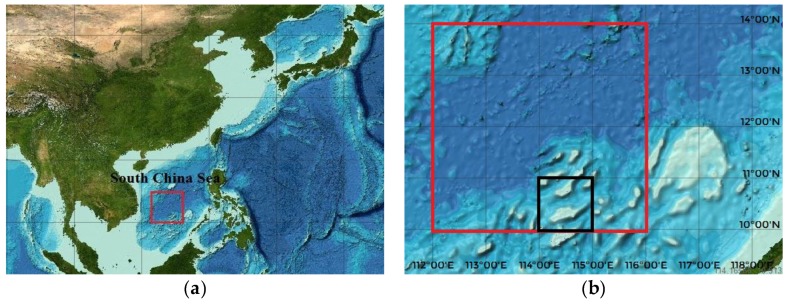
Satellite images of the study area: (**a**) location of study area; and (**b**) local amplification.

The resolution of original seabed digital terrain map was 0.5’ × 0.5’ in this experiment, which can be interpolated onto a grid with 100 × 100 m resolution, which provided a good base for us to use the terrain database for auxiliary navigation. The General Bathymetric Chart of the Oceans data was used in the study. The study area was located in the South China Sea (solid line box, Figure 7), ranging between longitude 114°–115° E and latitude 10°–11° N, as shown in Figure 7.

**Figure 8:**
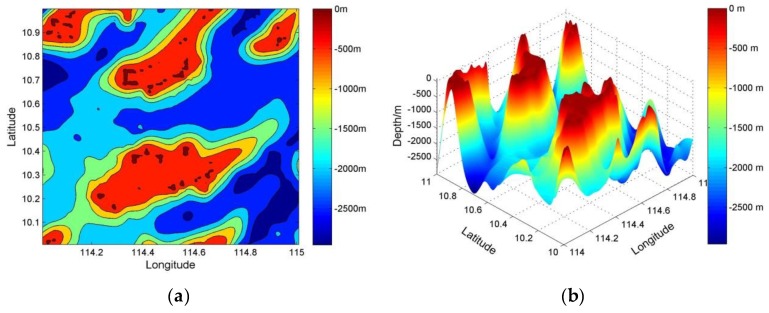
Seabed digital terrain map with a grid resolution of 100 × 100 m: (**a**) two-dimensional (2D); and (**b**) three-dimensional (3D) images.

Figure 8 shows the two-dimensional (2D)/three-dimensional (3D) seabed digital terrain map with a grid resolution of 100 × 100 m in the study area. Figure 8 is consistent with the satellite images in Figure 7. In Figure 8, many shallows and seamounts are distributed on the seabed, and terrain changes violently, all of which helped to set a simulation route.

**Figure 9:**
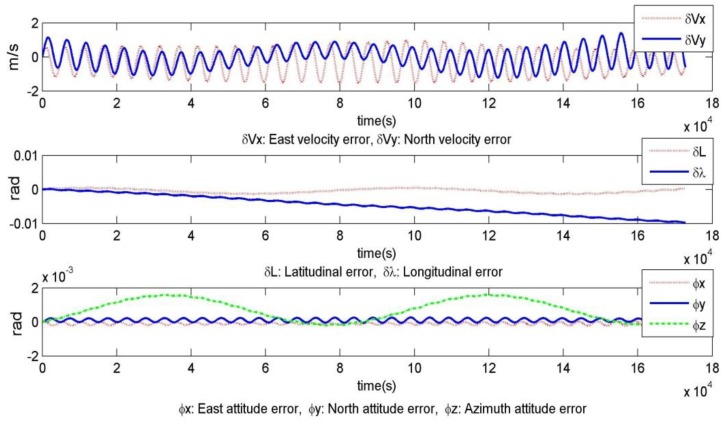
Estimation of the error divergence rule based on the stationary base.

We assumed that the underwater vehicle starts from position A and travels to B. Figure 9 shows the estimative curves of the error divergence, such as east/north velocity error, latitudinal/longitudinal error, and east/north/azimuth attitude error. The parameters used were as follows: gyroscope drift 0.01°/h, accelerometer zero bias 10^–3^ m/s^2^, latitude 10.25°, and running time 48 h.

**Figure 10:**
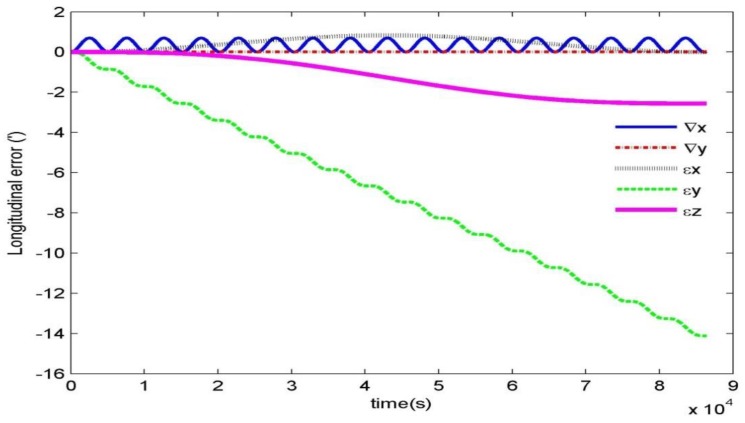
Influence of the gyroscope drifts and accelerometer zero bias on longitudinal error.

As shown in Figure 9, for the INS, the latitudinal error shows cyclical period changes and the longitudinal error accumulates over time. Figure 10 shows the influence of the gyroscope drifts (εx,εy,εz) and accelerometer zero bias (∇x,∇y) on longitudinal error. The impact analysis follows.

(1) The gyroscope drift causes accumulative error on the longitude over time, which is mainly caused by the north gyroscope drift (εy) and the azimuth gyroscope drift (εz). However, the east gyroscope drift (*εx*) does not cause accumulative error.

(2) The accelerometer zero bias does not cause accumulative error on the longitude over time.

**Figure 11:**
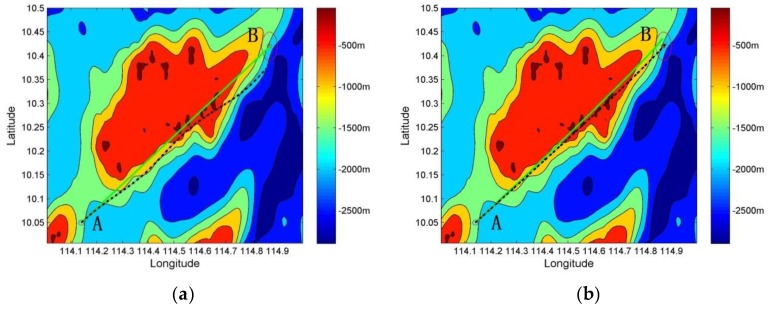
Schematic diagram between the inertial trajectory (dotted line) and the real trajectory (solid line) without underwater terrain matching navigation: (**a**) gyro 0.01°/h, accelerometer 10^−3^ m/s^2^; and (**b**) gyro 0.01°/h, accelerometer 10^−3^ m/s^2^, with geodesic-based method.

As shown in Figure 11, the parameters were set as follows: gyroscope drift 0.01°/h, accelerometer zero bias 10^−3^ m/s^2^, velocity 10 m/s, initial position error 0 m, velocity error 0.03 m/s, and period of geodesic-based method (T = 360 s).

Figure 11 compares the real-time error between the inertial trajectory and the real trajectory based on two approaches. Figure 11a shows the inertial trajectory (dotted line) and the real trajectory (solid line) of the underwater vehicle without the geodesic-based method. Figure 11b shows the inertial trajectory (dotted line) and the real trajectory (solid line) of the underwater vehicle based on the geodesic-based method.

**Figure 12:**
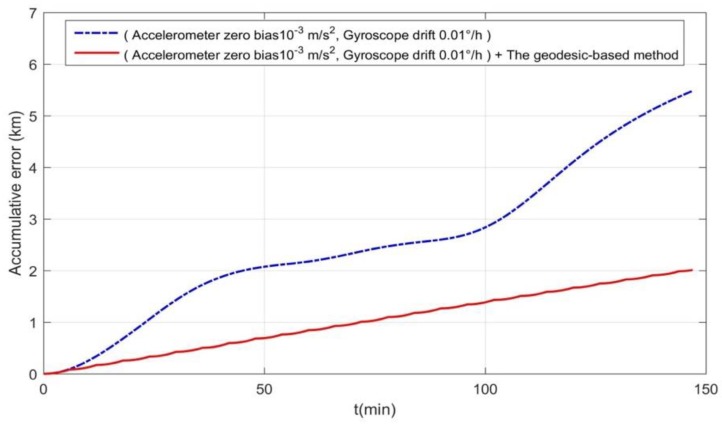
Comparison of the INS error and the error of INS + geodesic-based method.

Figure 12 shows that the navigation error between the inertial trajectory and the real trajectory is large (dotted line). However, the navigation error between the inertial trajectory and the real trajectory can be greatly reduced (solid line) based on the geodesic-based method with the same parameters (gyroscope drift 0.01°/h, accelerometer zero bias 10^−3^ m/s^2^). Therefore, the radius of the search matching area can be reduced and the matching efficiency can be improved.

### 3.2. Application of the Geodesic-Based Method

**Figure 13:**
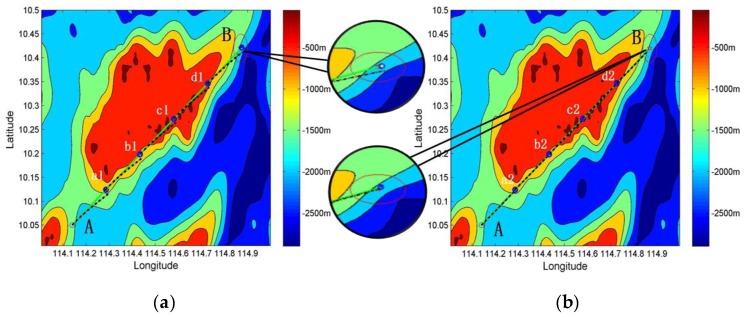
Schematic diagram of underwater terrain matching navigation (the dotted line displays the inertial trajectory, and the solid line displays the real trajectory): (**a**) underwater terrain matching navigation; and (**b**) underwater terrain matching navigation with geodesic-based method.

Figure 13 depicts a comparison of underwater terrain matching navigation based on the two approaches. The numerical simulation parameters of the TERCOM algorithm were set as follows: gyroscope drift 0.01°/h, accelerometer zero bias 10^−3^ m/s^2^, velocity 10 m/s, initial position error 0 m, velocity error 0.03 m/s, heading error 0.05°, number of sample points 110, and sampling period 10 s. The data of the sample point are the sum of the sampled value in the seabed digital terrain map and the random noise with a standard deviation of 20 m. The period of the geodesic-based method is T = 360 s.

We conducted 50 simulation experiments under the same conditions. As shown in Figure 13, the dotted line displays the inertial trajectory, the solid line displays the real trajectory, A is the starting point, and B is the ending point. In the process from A to B, there are four terrain matching points.

Figure 13a depicts the terrain matching map. When the underwater vehicle reached destination B, the navigation error between the inertial trajectory and the real trajectory was large. Without the geodesic-based method, the navigation error accumulates quickly over time. As shown in Figure 12 (dotted line), the accumulative navigation error was about 2 km/h.

Figure 13b shows the terrain matching map produced using the geodesic-based method. When the underwater vehicle reached destination B, the navigation error between the inertial trajectory and the real trajectory was smaller than that in Figure 13a. The heading angle was adjusted at every fixed time interval using the geodesic-based method based on INS. According to Figure 12 (solid line), the speed of accumulative navigation error has been restrained, helping to reduce the radius of the search matching area. Thus, the efficiency of search matching was improved.

## 4. Discussion

**Figure 14:**
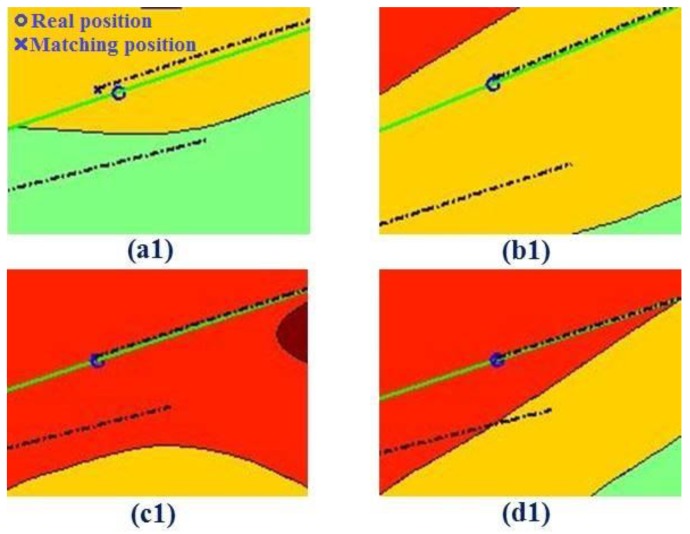
Schematic diagram of underwater terrain matching point (the dotted line displays the inertial trajectory, and the solid line displays the real trajectory).

Figure 14 depicts the schematic diagram of the terrain matching points, where the dotted line displays the inertial trajectory and the solid line displays the real trajectory (corresponding to Figure 13a). Table 1 lists the statistical results from Figure 14. According to a1, b1, c1, and d1 in Table 1, the circular error probable (CEP) before matching (the error between the inertial position and the real position) is large (about 1.3 km). Therefore, the search matching time is about 9.84 s. After matching, the matching position is better than the inertial position. As shown in Table 1 for b1, c1, and d1, the positioning accuracy (the error between the matching position and the real position) is within 100 m, and the effect of the positioning accuracy is better than for a1, which reaches 310.2 m. The suitability of terrain near the matching point a1 is slightly worse than that of the other three positions, and there may be many sets of bathymetric measurements with high similarity in the matching area.

**Figure 15:**
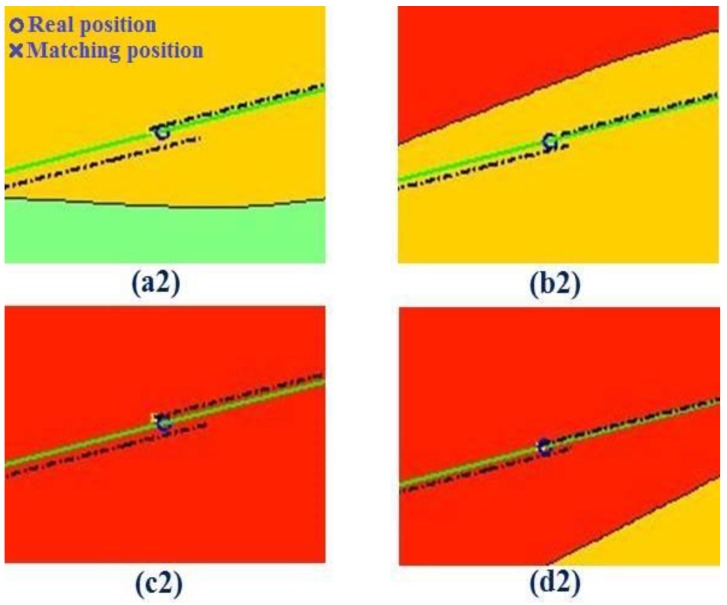
Schematic diagram of underwater terrain matching point based on the geodesic-based method (the dotted line displays the inertial trajectory, and the solid line displays the real trajectory).

Figure 15 shows the schematic diagram of the terrain matching points based on the geodesic-based method, where the dotted line displays the inertial trajectory and the solid line displays the real trajectory (corresponding to Figure 13b). Table 2 provides the statistical results from Figure 15. According to a2, b2, c2, and d2 in Table 2, the CEP before matching (the error between the inertial position and the real position) is small (about 500 m) due to the use of the geodesic-based method. Therefore, the search matching time is about 1.29 s. After matching, the matching position is better than the inertial position. As shown in a2, b2, c2, and d2 in Table 2, the positioning accuracy (the error between the matching position and the real position) is within 100 m and the stability is also better.

Comparing Table 1 and Table 2, due to the reduction of the radius of the search matching area, the search matching time is reduced from 9.84 s to 1.29 s, with the matching accuracy remaining stable using the new geodesic-based method. In other words, the search matching efficiency increases 7.6 times. The possibility of similar trajectories in the matching area is also reduced, and the matching probability is improved to a certain extent.

## 5. Conclusions

This paper proposed a new geodesic-based method to improve the matching efficiency of underwater terrain matching navigation.

(1) We developed a geodesic-based method by combining the law of the shortest arc in spherical geometry with the theory of the attitude control in space and maritime environments.

(2) We verified and applied the geodesic-based method. The computational complexity was decreased by reducing the radius of the search matching area. Therefore, the matching efficiency improved. For the parameter setting in this study, the search matching time of underwater terrain matching navigation was reduced from 9.84 s to 1.29 s (about 7.6 times), with accuracy being maintained using the new geodesic-based method.

(3) This study is not without its limitations and future work. The method proposed is an optimal heading planning method to the TERCOM algorithm. It can improve the matching efficiency by reducing the radius of the search matching area. Firstly, we only considered the case of a simple linear path and did not consider controlled heading in the case of a complex path. Secondly, we did not consider the influence of the position drift on heading that the TERCOM algorithm does not allow maneuvering during the matching process. Therefore, the above problems will be the focus our follow-up research work.

## Figures and Tables

**Table 1:** Statistical results from Figure 14.

	Information on Matching Point		Circular Error Probable (CEP) (m)		Search Matching Time
	Number	Coordinates	Before Matching	Positioning Accuracy	
Underwater terrain matching navigation	(a1)	10.123° N,114.287° E	1447.6	310.2	9.84 s
	(b1)	10.199° N,114.433° E	1631.5	111.6	
	(c1)	10.273° N,114.579° E	1252.6	102.9	
	(d1)	10.346° N,114.727° E	1174.6	44.6	

**Table 2:** Statistical results from Figure 15.

	Information on Matching Point		CEP (m)		Search Matching Time
	Number	Coordinates	Before Matching	Positioning Accuracy	
Underwater terrain matching navigation + geodesic-based method	(a2)	10.124° N,114.287° E	536.6	73.7	1.29 s
	(b2)	10.199° N,114.434° E	469.3	96.5	
	(c2)	10.274° N,114.579° E	560.6	86.9	
	(d2)	10.348° N,114.727° E	404.4	53.4	
